# The Role of NMDA Receptors in Alzheimer’s Disease

**DOI:** 10.3389/fnins.2019.00043

**Published:** 2019-02-08

**Authors:** Jinping Liu, Lirong Chang, Yizhi Song, Hui Li, Yan Wu

**Affiliations:** ^1^School of Medicine, Tsinghua University, Beijing, China; ^2^Department of Anatomy, Ministry of Science and Technology Laboratory of Brain Disorders, Beijing Institute for Brain Disorders, Capital Medical University, Beijing, China

**Keywords:** NMDA receptor, amyloid beta, memantine, Alzheimer’s disease, synaptic dysfunction

## Abstract

In Alzheimer’s disease (AD), early synaptic dysfunction is associated with the increased oligomeric amyloid-beta peptide, which causes NMDAR-dependent synaptic depression and spine elimination. Memantine, low-affinity NMDAR channel blocker, has been used in the treatment of moderate to severe AD. However, clear evidence is still deficient in demonstrating the underlying mechanisms and a relationship between NMDARs dysfunction and AD. This review focuses on not only changes in expression of different NMDAR subunits, but also some unconventional modes of NMDAR action.

## Introduction

Alzheimer’s disease (AD) is an age-related neurodegenerative disease. Clinically, this disorder is characterized by global cognitive dysfunction, especially memory loss, behavior and personality changes. AD progression has been associated with a gradual damage in function and structure in the hippocampus and neocortex, the vulnerable brain areas used for memory and cognition (Mota et al., 2014). The neuropathologic hallmarks of AD include extracellular amyloid plaques, being composed of the amyloid beta (Aβ) peptide and intraneuronal neurofibrillary tangles (NFTs), consisting of abnormally hyperphosphorylated tau protein (Ferreira et al., 2010). Nevertheless, damage and destruction of synapses seem to better correlate with memory loss than histopathologic markers. Synapse loss can be caused by the failure of live neurons to maintain functional axons and dendrites or by neuron death (Bloom, 2014; Avila et al., 2017; Morris et al., 2018).

Synaptic dysfunction may be due to perturbed synaptic Ca^2+^ handling in response to over activation of glutamate receptors, namely, the *N*-methyl-D-aspartate receptors (NMDARs) (Mota et al., 2014). Glutamate is the primary excitatory neurotransmitter in the brain, acting at ionotropic and metabotropic glutamate receptors. Ionotropic glutamate receptors (iGluRs), responsible for fast neuronal communication at excitatory synapses, comprise three subfamilies: α-amino-3-hydroxy-5-methyl-4-isoxasolepropionic acid (AMPA) receptors, kainate receptors, and NMDARs (Traynelis et al., 2010). However, excessive stimulation of glutamatergic signaling results in excitotoxicity (Rothman and Olney, 1986). Besides acute effects, many studies indicate a role for glutamate excitotoxicity in delayed slowly evolving neurodegeneration (Choi, 1988; Epstein et al., 1994). The toxicity is principally mediated by excessive Ca^2+^ entry, primarily through NMDARs (Choi, 1987; Tymianski et al., 1993), since NMDARs have a much higher permeability for calcium ions compared to other iGluRs (Choi, 1992; Wang and Reddy, 2017).

*N*-methyl-D-aspartate receptor plays a pivotal role in the synaptic transmission and synaptic plasticity thought to underlie learning and memory, which is not only central to the development and function of the nervous system, but also to neurotoxicity. NMDAR activation has been recently implicated in AD related to synaptic dysfunction (Paoletti et al., 2013; Kodis et al., 2018). The pathological level of Ca^2+^ signaling leads to gradual loss of synaptic function and ultimate neuronal cell death, which correlates clinically with the progressive decline in cognition/memory and the development of pathological neural anatomy seen in AD patients. And this, in turn, rationalizes the clinical trial of memantine, an NMDAR antagonist, as a symptomatological and neuroprotective treatment for AD (Danysz and Parsons, 2003; Wenk, 2006; Wang and Reddy, 2017). Memantine, a non-competitive NMDA receptor antagonist, is approved for use in moderate to severe AD. It has been widely prescribed to provide symptomatic relief and enhance life quality in AD, even if it did not improve excessive agitation (Fox et al., 2012), and hippocampal or total brain atrophy (Wilkinson et al., 2012). However, in the brain areas mainly affected in AD, NMDARs are mainly composed by GluN2A and GluN2B subunits. Furthermore, taking into account the fact that extrasynaptic GluN2B-containing NMDARs have been associated with excitotoxicity in AD (Hardingham and Bading, 2010), the use of a selective GluN2B subunit antagonists might be an interesting strategy to prevent synaptic dysfunction in AD.

## NMDA Receptors

*N*-methyl-D-aspartate receptor is a ligand of glutamate, which is the primary excitatory neurotransmitter in the human brain. Most NMDAR subtypes are unique in that their opening requires the coincidence of both presynaptic glutamate release and a strong postsynaptic membrane depolarization to relieve Mg^2+^ block of the channel (Mayer et al., 1984; Nowak et al., 1984). NMDARs are permeable to Na^+^, K^+^, and high permeability to Ca^2+^, which acts as a second messenger to modify synapses (Lynch et al., 1983). NMDARs are essential mediators of brain plasticity and are capable of converting specific patterns of neuronal activity into long-term changes in synapse structure and function that are thought to underlie higher cognitive functions (Traynelis et al., 2010). Activation of NMDARs leads to cytosolic free intracellular calcium ([Ca^2+^]_i_) increase (MacDermott et al., 1986), required for long-term potentiation (LTP) and long-term depression (LTD) (Muller et al., 2009), and, more generally, for synaptic plasticity (MacDonald et al., 2006; Lau et al., 2009). NMDARs are also thought to be involved in a process called excitotoxicity. Abnormal NMDAR activity is associated with seizure, ischemic stroke (Choi et al., 1988; Villmann and Becker, 2007), and neurodegenerative disorders (Benarroch, 2011), such as Alzheimer’s (Wenk, 2006), Huntington’s (Fan and Raymond, 2007), and Parkinson’s disease (Bonuccelli and Del Dotto, 2006).

*N*-methyl-D-aspartate receptors are also expressed across a wide spectrum of non-neuronal cells, including central and peripheral glial cells, endothelium, bone, kidney, pancreas, and others (Hogan-Cann and Anderson, 2016). Astrocytes express functional NMDARs capable of responding to neuronal glutamatergic input (Dzamba et al., 2013) and neuroinflammatory processes (Sofroniew, 2009; Ting et al., 2009). Potential roles for endothelial NMDARs is in the area of blood–brain barrier (BBB) function. Uncontrolled glutamate levels in brain are toxic to neurons, damage endothelial function and disrupt BBB integrity (András et al., 2007; Basuroy et al., 2013). Osteoblast NMDARs stimulated precursor differentiation and contributed to increased bone mineralization, leading to bone matrix deposition (Hinoi et al., 2003; Li et al., 2011). Renal NMDAR activity stimulated vasodilation in the glomerulus, thereby influencing renal blood flow, filtration, and reabsorption in the proximal tubule (Deng and Thomson, 2009; Anderson et al., 2011; Sproul et al., 2011). In the pancreas, NMDARs are expressed by insulin-producing islet β cells, and influence β cell function and survival (Inagaki et al., 1995; Molnár et al., 1995; Marquard et al., 2015). In the lung, airway smooth muscle cells express NMDARs, which may be involved in inflammatory bronchiole hyper-reactivity (Antošová and Strapková, 2013; Anaparti et al., 2015).

Seven different NMDAR subunits have been identified: the GluN1 subunit, four distinct GluN2 subunits (GluN2A, GluN2B, GluN2C, and GluN2D), and a pair of GluN3 subunits (GluN3A and GluN3B) (Paoletti, 2011). Functional NMDARs are heterotetramers composed of two glycine or D-serine-binding GluN1 subunits and two glutamate-binding GluN2 (GluN2A-D) subunits or, in some cases, glycine-binding GluN3 (GluN3A/B) subunits (Köhr, 2006). All NMDAR subunits share a common membrane topology that contain four discrete domains (Lee et al., 2014; Karakas and Furukawa, 2014): The extracellular amino-terminal domain (ATD) is involved in subunit assembly and allosteric regulation, and contributes to control of ion channel open probability and deactivation speeds. The extracellular ligand-binding domain (LBD) is formed by two discontinuous segments (S1 and S2), and binds agonists and antagonists to control ion channel opening. The transmembrane domain (TMD) contains three transmembrane segments (M1, M3, and M4) and a re-entrant pore loop (M2) which is part of the channel pore, containing a critical asparagine residue that determines calcium permeability of the channel and mediates the magnesium blockade (Paoletti et al., 2013; Karakas and Furukawa, 2014). The intracellular carboxyl-terminal domain (CTD) interacts with multiple cytosolic proteins (Sanz-Clemente et al., 2012). GluN2 LBDs contain the glutamate binding site, while GluN1 (or GluN3) LBDs contain the Glycine/D-serine coagonist binding site (Hackos and Hanson, 2017). In general, activation of NMDARs containing GluN1/GluN2 requires binding of both glycine and glutamate at the extracellular LBDs in addition to release of magnesium block by membrane depolarization at the TMD (Regan et al., 2015). Positively charged residues on the surface of the GluN2A LBD assist glutamate binding via a ‘guided-diffusion’ mechanism. On the other hand, Glycine binds to the GluN1 LBD via an ‘unguided-diffusion’ mechanism, whereby glycine finds its binding site primarily by random thermal fluctuations (Yu and Lau, 2018). A recent study show, through cryoelectron microscopy, that the presence of the exon 5 motif, at the ATD of the GluN1 subunit, alters the local architecture of heterotetrameric GluN1/GluN2 NMDA receptors and creates contacts with the LBDs of the GluN1 and GluN2 subunits. The unique interactions established by the exon 5 motif are essential to the stability of the ATD/LBD and LBD/LBD interfaces that are critically involved in controlling proton sensitivity and deactivation (Regan et al., 2018). The most widely expressed NMDARs contain the obligatory subunit GluN1 plus either GluN2B or GluN2A or a mixture of the two; therefore, in next sections we will focus on these two subunits.

## NMDA Receptors in AD

The defining features of AD include conspicuous changes in both brain histology and behavior. AD brain is characterized microscopically by extracellular amyloid plaques and intraneuronal NFTs. Accumulating evidence indicated that soluble forms of Aβ and tau work together, independently of their accumulation into plaques and tangles, to drive healthy neurons into the diseased state and that hallmark toxic properties of Aβ require tau (Bloom, 2014). The cognitive impairment of AD is closely related to synaptic plasticity, in which NMDAR plays a critical role (Parameshwaran et al., 2008). Excitatory glutamatergic neurotransmission via NMDAR is critical for synaptic plasticity and survival of neurons. However, excessive NMDAR activity causes excitotoxicity and promotes cell death, underlying a potential mechanism of neurodegeneration occurred in AD (Wang and Reddy, 2017). The major factors that affect NMDAR signaling in AD include glutamate availability and the modulation of NMDAR channel functions (Wang and Reddy, 2017).

### Aβ and NMDA Receptors

#### Aβ in AD

In AD, Aβ cascade hypothesis posits an initiating event of amyloidosis, with subsequent tau accumulation preceding downstream brain atrophy and cognitive decline (Hardy, 2002). From amyloid-PET and florbetapir-PET studies, it has been known that Aβ deposition occurs selectively first in the cortex, beginning in temporobasal and frontomedial areas, and successively affecting primary sensory-motor areas and the medial temporal lobe. It was followed by hippocampal regions, then striatum, basal forebrain, thalamus, and finally brainstem nuclei and cerebellum (Grothe et al., 2017; Rice and Bisdas, 2017; Whitesell et al., 2018). Deposition of Aβ in the medial parietal cortex appears to be the first stage in the development of AD, although tau aggregates in the medial temporal lobe precede Aβ deposition in cognitively healthy older people (Jagust, 2018).

Aβ is produced by endoproteolysis of the parental amyloid precursor protein (APP), which is achieved by the sequential cleavage of APP by groups of enzymes termed β- and γ-secretases (LaFerla et al., 2007). Aβ is produced as a monomer, but readily aggregates to form multimeric complexes. Human Aβ can exist in diverse assembly states, including monomers, dimers, trimers, tetramers, dodecamers, higher-order oligomers and protofibrils, as well as mature fibrils, which can form microscopically visible amyloid plaques in brain tissues (Glabe, 2008). The original amyloid hypothesis postulated that accumulation of Aβ in the brain is the primary influence driving AD pathogenesis. Cell studies and animal experiments confirmed that oligomeric, soluble Aβ rather than insoluble amyloid plaques exert the toxic effect (Lacor et al., 2007; Shankar et al., 2007). Thus, according to the modified amyloid cascade hypothesis, soluble oligomeric assemblies of Aβ induce the neurodegenerative triad (Wu et al., 2010).

The Aβ peptide was first identified as a component of extracellular amyloid plaques in the mid-1980s. Now, there have been a large number of studies have provided evidence for the presence of intracellular Aβ within neurons (LaFerla et al., 2007). Immunogold electron microscopy has been carried out to demonstrate that Aβ_1-42_ can be found in multivesicular bodies (MVBs) of neurons in the human brain, where it is associated with synaptic pathology. Studies also show that Aβ produced intracellularly in the endosome compartments (Kinoshita, 2003; Rogaeva et al., 2007), ER (endoplasmic reticulum) (Busciglio et al., 1993; Cook et al., 1997), and trans-Golgi network in neurons (Hartmann et al., 1997). The secreted Aβ, which forms the extracellular Aβ pool, could be taken up by cells and internalized into intracellular pools. Aβ binds to the α7 nicotinic acetylcholine receptor (α7nAChR) with high affinity, resulted in receptor internalization and accumulation of Aβ intracellularly (Wang et al., 2000; Nagele et al., 2002). In addition to nicotinic receptors, Aβ internalization has been reported through LRP (low-density lipoprotein receptor related protein) (Bu et al., 2006), RAGE (scavenger receptor for advanced glycation end products) (Deane et al., 2003), and NMDAR (Caldeira et al., 2013). Aβ uptake was completely blocked by NMDAR antagonists, evidencing an involvement of this receptor in the re-uptake of the peptide (Bi et al., 2002).

Aβ oligomerization initiates within cells rather than in the extracellular space (Walsh et al., 2000). Much evidence suggests that Aβ oligomers are more potent than Aβ fibrils in eliciting abnormalities in synaptic functions. Moreover, oligomers of Aβ_1-42_ are considered the most synaptotoxic forms, responsible for early cognitive deficits in AD (Palop and Mucke, 2010). Aβ leads to aberrant excitatory network activity and compensatory inhibitory responses involving learning and memory circuits (Palop et al., 2007). Intracellular Aβ within the MVBs is mechanistically linked to cytosolic proteasome inhibition, lead to higher Aβ levels and the buildup of tau protein (Almeida, 2006; LaFerla et al., 2007). Low (picomolar range) concentrations of Aβ markedly potentiate synaptic transmission, whereas higher concentrations (low nanomolar range) of Aβ cause synaptic depression by acting on α7nAChR activation (Dineley et al., 2002; Puzzo et al., 2008).

Accumulation of intracellular Aβ in mitochondria is associated with the multitude of mitochondrial defects described in AD (Manczak et al., 2006). Aβ is transported into mitochondria via the translocase of the outer membrane machinery and is located in mitochondria cristae (Hansson Petersen et al., 2008). In AD postmortem brain and cellular and animal AD models, mitochondrial dysfunction can be triggered by Aβ. Aβ-induced mitochondrial dysfunction was reported to be related to the interaction of Aβ with different mitochondrial proteins, including proteins of the outer mitochondrial membrane, intermembrane space, inner mitochondrial membrane, and the matrix, impairing oxidative phosphorylation and mitochondrial dynamics and increasing reactive oxygen species (ROS) production (Pagani and Eckert, 2011).

Aβ also induce ER stress. In mature hippocampal cultures, Aβ induce NADPH oxidase (NOX)-mediated superoxide production downstream of GluN2B and impairs ER and cytosolic Ca^2+^ homeostasis (Costa et al., 2012). In rat brain endothelial cells (RBE4 cell line), Aβ increased the levels of several markers of ER stress-induced unfolded protein response (UPR), affected the Ca^2+^ homeostasis (Fonseca et al., 2013). In turn, ER stress mediated Aβ production or neurotoxicity. Increased the ER stress promoted Aβ production was observed in RGC-5 cells, a cell line identical to the photoreceptor cell line 661W (Liu et al., 2014). Knockdown of syntaxin 5 expression enhanced the secretion of Aβ peptides under condition without ER stress in NG108-15 cells (Suga et al., 2015). In PC12 cells, the ER stress response factor X-box binding protein 1 (XBP1) prevents Aβ-introduced accumulation of [Ca^2+^]_i_ (Casas-Tinto et al., 2011).

Aβ was shown to be produced in high amounts in synaptic terminals of the hippocampal dentate gyrus of transgenic mice and deposited in extracellular plaques (Lazarov et al., 2002). Indeed, studies in AD postmortem brain tissue samples and AD animal models support a role for disruption of synaptic Ca^2+^ regulation in the neurotoxic action of Aβ, which may serve as a trigger for synaptic deterioration driving the cognitive loss in AD (Camandola and Mattson, 2011; Mota et al., 2014). Cognitive function ultimately depends on synaptic plasticity where LTP is associated with synapse growth and LTD is associated with synapse loss. Aβ is associated with the inhibition of LTP (Walsh et al., 2002) and the promotion of LTD (Li et al., 2009; Hunter et al., 2018). During the induction of LTP, the strong and prolonged release of glutamate from the presynaptic terminal activates AMPA receptors and the subsequent depolarization removes the Mg^2+^ blockade of the NMDAR channel and allows the influx of Ca^2+^. This strong activation of NMDARs triggers a Ca^2+^/calmodulin-dependent protein kinase II (CaMKII)-mediated signaling cascade that eventually leads to an enhanced synaptic strength. On the contrary, a modest activation of NMDARs causes a modest increase in postsynaptic Ca^2+^ and triggers phosphatases-mediated LTD (Luscher and Malenka, 2012). Activation of synaptic NMDARs and large increases in [Ca^2+^]_i_ are required for LTP, whereas internalization of synaptic NMDARs, activation of perisynaptic NMDARs and lower increases in [Ca^2+^]_i_ are necessary for LTD. LTP induction promotes recruitment of AMPA receptors and growth of dendritic spines, whereas LTD induces spine shrinkage and synaptic loss (Kullmann and Lamsa, 2007). Pathologically elevated Aβ may indirectly cause a partial block of NMDARs and shift the activation of NMDAR-dependent signaling cascades toward pathways involved in the induction of LTD and synaptic loss (Kamenetz et al., 2003; Shankar et al., 2007). This model is consistent with the fact that Aβ impairs LTP (Walsh et al., 2002; Cleary et al., 2005) and enhances LTD (Kim et al., 2001; Hsieh et al., 2006; Li et al., 2009). Although the mechanisms underlying Aβ-induced LTD have not yet been fully elucidated, they may involve receptor desensitization (Liu, 2004) or internalization and subsequent collapse of dendritic spines (Snyder et al., 2005; Hsieh et al., 2006).

Aβ modulated NMDA-induced responses and vice versa; pre-exposure to Aβ decreased NMDA-evoked [Ca^2+^]_i_ rise and pre-exposure to NMDA decreased Aβ response. In addition, simultaneous exposure to Aβ plus NMDA synergistically increased [Ca^2+^]_i_ levels, an effect mediated by GluN2B-containing NMDARs (Ferreira et al., 2012). Accumulating evidence demonstrates that glutamate receptors are dysregulated by Aβ oligomers, resulting in disruption of glutamatergic synaptic transmission which parallels early cognitive deficits. In theory, there are several potential roles for the NMDA receptor in Aβ-related mechanisms (Malinow, 2012): first, NMDA receptor function may be an important downstream target of Aβ; second, NMDA receptors may be necessary in the actions of Aβ on synaptic transmission and plasticity; third, NMDA receptor may be a receptor for Aβ, or indirectly; and fourth, NMDA receptor activity may control the formation of Aβ.

#### The Effect of Aβ on NMDAR Subunits

Expression of NMDAR subunits differentially distribute throughout the brain and change strikingly during development. The glycine/D-serine binding GluN1 subunit is an obligatory subunit in all NMDA receptor subtypes. In accordance with the widespread central nervous system (CNS) distribution of NMDARs, the GluN1 subunit is ubiquitously expressed from embryonic stage E14 to adulthood (Monyer et al., 1994). The four different glutamate binding GluN2 subunits (GluN2A-D) have pronounced differences in both developmental and regional expression levels and endow NMDA receptors with strikingly different pharmacological and functional properties. In the embryonic brain, only GluN2B and GluN2D subunits are expressed, and the latter is mostly found in caudal regions. During the first two postnatal weeks, GluN2A expression rises steadily to become widely and abundantly expressed in virtually every CNS area in the adult. Meantime, GluN2D expression drops markedly, and in the adult, it is expressed at low levels mostly in the diencephalon and mesencephalon. GluN2B expression is maintained at high levels following birth, peaks around the first postnatal week and becomes progressively restricted to the forebrain. Expression of GluN2C appears late in development, about postnatal day 10, and mainly confined to the cerebellum and the olfactory bulb (Paoletti et al., 2013).

In the adult CNS, particularly in higher brain structures, such as the hippocampus and cortex, GluN2A and GluN2B are the predominant subunits, indicating that they have central roles in synaptic function and plasticity (Monyer et al., 1994; Takai et al., 2003). GluN2C- or GluN2D- containing receptors appear to give rise to ‘low-conductance’ openings with a lower sensitivity to extracellular Mg^2+^, which may affect the Ca^2+^ influx generated by synaptic activation of NMDAR (Momiyama et al., 1996). Misra et al. (2000) results indicate that GluN1/GluN2D receptors do not contribute to the excitatory postsynaptic current (EPSC) and appear to be restricted to the extrasynaptic membrane. The possibility still exists that the GluN2D subunit is present at the synapse but is preferentially co-assembled with other GluN2 subunits, such as triheteromeric assemblies (GluN1/GluN2B/GluN2D) (Dunah et al., 1998; Cull-Candy et al., 2001). GluN3A and GluN3B were cloned based on similarity to GluN1 and GluN2 subunits and were the last NMDA receptor subunits to be discovered (Low and Wee, 2010). GluN3A expression is low embryonically, peaks during early postnatal life, and diminishes to lower levels in adulthood. This expression profile is observed in many regions of the brain (Bendová et al., 2009; Roberts et al., 2009). Conversely, GluN3B levels are low around postnatal day 10 and gradually increase into adulthood within the neocortex, hippocampus, striatum, cerebellum, brainstem, and spinal cord (Wee et al., 2008). GluN3A and GluN3B are expressed by multiple neuronal cell types, including interneurons, pyramidal cells, motor neurons, trigeminal neurons, retinal ganglion and amacrine cells (Pachernegg et al., 2012). Although GluN3A is present in oligodendrocytes, it does not seem to be expressed in astrocytes (Matsuda et al., 2003; Káradóttir et al., 2005; Salter and Fern, 2005). The GluN3 subunits bind glycine and D-serine (Yao, 2006; Yao et al., 2008), but the functional properties and physiological roles of GluN3-containing NMDA receptors remain elusive. GluN3A acts in a dominant-negative manner to suppress receptor activity. GluN3A containing NMDARs display striking regional and temporal expression specificity, and, unlike most other NMDAR subtypes, they have a low conductance, are only modestly permeable to Ca^2+^, and pass current at hyperpolarized potentials in the presence of magnesium. While glutamate activates triheteromeric NMDARs composed of GluN1/GluN2/GluN3A subunits, glycine is sufficient to activate diheteromeric GluN1/GluN3A-containing receptors (Henson et al., 2010). About GluN3A is expressed in the right place at the right time to regulate spine and synapse development, there are two hypotheses, if the influence of GluN3A is to serve as a ‘synaptic brake’ to limit synapse/spine formation or if it serves as a ‘synaptic marker’ to promote the elimination of spines (Henson et al., 2010). Roberts et al. (2009) found that prolonging GluN3A expression prevents glutamatergic synapse maturation by limiting synapse potentiation and growth, and decreasing spine density, accompanied by major impairments in learning and memory processes. Whereas knocking out endogenous GluN3A conversely accelerates synaptic maturation events.

In line with the large number of subunits and their overlapping expression in several brain regions, many different NMDAR subtypes coexist in the CNS. All NMDAR subtypes are thought to combine two copies of the obligatory GluN1 subunit plus two copies of GluN2 and/or GluN3 subunits. Diheteromeric GluN1/GluN2B and GluN1/GluN2A receptors represent an important fraction of juvenile and adult NMDARs. Triheteromeric GluN1/GluN2A/GluN2B receptors also populate many regions in the adult brain, particularly in the hippocampus and cortex, with estimates of abundance ranging from 15% to >50% of the total receptor population (Al-Hallaq et al., 2007). They may maintain some signaling properties of the GluN2B subunit while having the kinetic properties of GluN1/2A NMDARs (Sun et al., 2017). The sensitivity of GluN1/GluN2A/GluN2B triheteromers to the agonists, glutamate and glycine, is intermediate to that of diheteromers. The sensitivity to glycine, conferred by the GluN1 sub-units, is close to that of GluN1/GluN2B receptors, whereas the sensitivity to glutamate, conferred by the GluN2 sub-units, is closer to that of GluN1/GluN2A receptors (Hansen et al., 2014; Stroebel et al., 2014). Triheteromeric GluN1/GluN2A/GluN2C receptors and GluN1/GluN2B/GluN2D receptors have also been described. Triheteromeric GluN1/GluN2A/GluN2C receptors are the predominant NMDARs in cerebellar granule cells and propose that co-expression of GluN2A and GluN2C in cerebellar granule cells occludes cell surface expression of diheteromeric GluN1/GluN2C receptors. For many agonists, potentiators, and inhibitors as well as endogenous ligands, GluN1/GluN2A/GluN2C NMDARs had intermediate properties compared with diheteromeric receptors (Bhattacharya et al., 2018). Similarly, triheteromeric GluN1/GluN2A/GluN2D and GluN1/GluN2B/GluN2D complexes populate the rat thalamus and midbrain (Dunah et al., 1998), as well as the human spinal cord (Sundström et al., 1997). GluN3A and GluN3B subunits can co-assemble with GluN2 subunits to form triheteromeric GluN1/GluN2/GluN3 receptors that are involved in synapse maturation during brain development (Paoletti, 2011; Paoletti et al., 2013; Pérez-Otaño et al., 2016).

*N*-methyl-D-aspartate receptors dysregulation evoked by Aβ and the consequent loss of Ca^2+^ homeostasis are thought to be related to the early cognitive deficits observed in AD. Many researches have demonstrated that Aβ oligomers and NMDAR contribute to the synaptic dysfunction in AD (Roselli et al., 2005; Liu et al., 2010; Chang et al., 2016; Li et al., 2016). The effect of Aβ on synapses is to produce depression of glutamatergic synaptic transmission (Kamenetz et al., 2003) and reduce surface glutamate receptors and other synaptic components in cultured hippocampal neurons (Snyder et al., 2005; Liu et al., 2010). NMDAR antagonist D-VAP can blocked the Aβ-induced depression of glutamatergic transmission (Kamenetz et al., 2003; Parameshwaran et al., 2008). Aβ accumulation may activate NMDARs at early stages of AD (Parameshwaran et al., 2008), and that Aβ oligomers can evoke an immediately [Ca^2+^]_i_ rise through activation of GluN2B-containing NMDARs in cultured cortical neurons (Ferreira et al., 2012). Conversely, Aβ-induced activation of GluN2A-containing NMDARs in non-neuronal cells, namely in *Xenopus laevis* oocytes (Texidó et al., 2011) and HEK293cells (Domingues et al., 2007), was previously reported.

The effects of Aβ on the mRNA and protein levels of NMDAR subunits have been extensively studied *in vivo* and *in vitro*, although the alterations of the NMDARs remain inconclusive. Some reported that levels of both GluN1 mRNA (Ułas and Cotman, 1997; Hynd et al., 2001) and GluN1 protein (Sze et al., 2001; Jacob et al., 2007) were significantly lower in AD patients. Conversely, other studies evidenced that levels of GluN1 mRNA were unchanged (Bi and Sze, 2002; Hynd et al., 2004). Moreover, levels of GluN2A and GluN2B mRNA were decreased in AD brain (Hynd et al., 2004), especially in hippocampus and the entorhinal cortex of postmortem (Bi and Sze, 2002). Whereas levels of GluN2C and GluN2D mRNA did not differ from that in controls (Hynd et al., 2004). The levels of GluN2A and GluN2B protein were also decreased in AD brain (Sze et al., 2001; Hynd et al., 2004). Other study found that mRNA expression and protein levels for GluN1 and GluN2B were significantly reduced, while GluN2A mRNA expression and protein levels were unchanged in hippocampus (Mishizen-Eberz et al., 2004).

Costa et al. (2012) data show that Aβ oligomers binding to mature hippocampal cells was prevented when the extracellular termini of GluN1 and GluN2B, but not of GluN2A, was blocked, indicating that Aβ oligomers are able to bind NMDARs extracellularly. These were consistent with the results that NMDARs may be direct or indirect targets for Aβ, by the obligatory GluN1 subunit, affecting the activity of these receptors (De Felice et al., 2007; Malinow, 2012). In the AD brain and human cortical neurons, excitatory synapses containing the GluN2B subunit of the NMDARs appear to be the main sites of Aβ oligomers accumulation. In addition, some studies report that Aβ oligomers may bind to EphB2, a surface tyrosine kinase that binds the NMDARs for maintaining its integrity. EphB2 degradation results in a decrease in surface localization of NMDA receptor subunits like GluN2B (Shi et al., 2016), the loss of NMDAR function and reduced LTP (Dalva et al., 2000).

Some results suggest a circuit in which Aβ facilitates NMDARs activation, which in turn control the formation of Aβ. NMDARs activation (Lesne, 2005), and more particularly extrasynaptic activation (Bordji et al., 2010), increased production and secretion of Aβ in primary cultures of cortical neurons, which was preceded by a shift from APP695 to Kunitz protease inhibitory domain (KPI) containing amyloid-β precursor protein (KPI-APP), isoforms exhibiting an important high amyloidogenic potential, followed by a shift from α-secretase to β-secretase-mediated APP processing (Hoey et al., 2009). An *in vivo* study indicates that the level of NMNDAR activation may control the effect on Aβ production: low levels of NMDAR activation increase Aβ production while higher levels reduce Aβ production (Verges et al., 2011). However, a study using *in vivo* microdialysis techniques demonstrated that the neuronal activity dependence of Aβ secretion lies in the presynaptic compartment suggesting no NMDAR dependence (Cirrito et al., 2005). The role of NMDAR in production of Aβ is likely complex and requires more investigation.

*N*-methyl-D-aspartate receptors mediate many forms of synaptic plasticity. These tetrameric receptors consist of two obligatory GluN1 subunits and two regulatory subunits, usually a combination of GluN2A and GluN2B. Among the six regulatory subunits of NMDARs, GluN2A and GluN2B have been the most extensively studied because they are broadly expressed in the brain, predominate in the postnatal cortex, and are believed to play important roles in synaptic plasticity. In the neonatal neocortex GluN2B-containing NMDARs predominate, and sensory experience facilitates a developmental switch in which GluN2A levels increase relative to GluN2B (Yashiro and Philpot, 2008). However, GluN1/GluN2B carry about twofold more charge for a single synaptic event than GluN1/GluN2A channels (Erreger et al., 2005). More studies showed that GluN2A-dominated synapses are more likely to induce LTD than GluN2B-dominated synapses, while GluN2B-dominated synapses have a greater capacity to be potentiated. GluN2A co-immunoprecipitates with neuronal nitric oxide (NO) synthase more effectively than GluN2B (Al-Hallaq et al., 2007). Although this interaction is likely indirect, the association raises the interesting possibility that NO-mediated presynaptic forms of LTP and LTD may be preferentially linked to GluN2A-mediated signaling pathways (Haghikia et al., 2007). Activated CaMKII binds strongly to GluN2B, which is required for LTP induction (Barria and Malinow, 2005). However, both GluN2A-containing and GluN2B-containing NMDARs are capable of supporting bidirectional synaptic plasticity (Yashiro and Philpot, 2008). GluN2A and GluN2B are present as either diheteromers (GluN1/GluN2A or GluN1/GluN2B) or triheteromers (GluN1/GluN2A/GluN2B) (Yashiro and Philpot, 2008). In isolated GluN2A-only or GluN2B-only synapses, GluN1/GluN2A diheteromeric channels exhibit faster rising and decaying currents than GluN1/GluN2B diheteromeric channels (Prybylowski et al., 2002). GluN1/GluN2A/GluN2B triheteromeric channels appear to exhibit intermediate decay time courses between the two diheteromeric channel types (Vicini et al., 1998).

Proteins interacting with NMDAR subunits are therefore important determinates for the direction of synaptic plasticity. The differential interaction of GluN2A and GluN2B subunits to membrane-associated guanylate kinase (MAGUKs) is controversial. It was once believed that GluN2A preferentially bound to postsynaptic density protein-95 (PSD-95), while GluN2B preferentially bound synapse-associated protein 102 (SAP102) (Sans et al., 2000; Krapivinsky et al., 2003). GluN2B interacts directly with Ras-guanine nucleotide releasing factor 1 (Ras-GRF1) (Krapivinsky et al., 2003), and synaptic Ras GTPase activating protein (RasGAP), presumably through SAP102 (Kim et al., 1998). The unique associations are likely to affect the induction of plasticity (Zhu et al., 2002). Moreover, these interactions were thought to control distinct synaptic localization of GluN2A and GluN2B (Townsend et al., 2003). However, a recent biochemical study using a serial immunoprecipitation suggests that MAGUK proteins such as PSD-95 and SAP102 interact with diheteromeric GluN1/GluN2A and GluN1/GluN2B receptors at comparable levels (Al-Hallaq et al., 2007). Additional studies are needed to clarify the association of NMDAR subunits with MAGUK family members and what effects these associations may have on receptor localization and on plasticity signaling pathways (Yashiro and Philpot, 2008).

Our results suggested that enhancement of GluN2A activity and/or the reduction of GluN2B activity may be used in order to halt the early Aβ-mediated synaptic dysfunction (Liu et al., 2010). GluN2A and GluN2B subunits have opposite roles in regulating [Ca^2+^]_i_ homeostasis. In rat cerebral cortical neurons, Aβ, like NMDA, increase [Ca^2+^]_i_ levels mainly through activation of GluN2B. Conversely, GluN2A antagonism potentiates [Ca^2+^]_i_ rise induced by a high concentration of Aβ (1 μM) (Ferreira et al., 2012). Simultaneous exposure to Aβ and NMDA affects the response to GluN2B-composed NMDARs, largely causing mitochondrial depolarization and mitochondrial Ca^2+^ (mitCa^2+^) retention (Ferreira et al., 2015). Aβ-induced ER stress and hippocampal dysfunction were prevented by ifenprodil, an antagonist of GluN2B subunits, while the GluN2A antagonist NVP-AAM077 only slightly attenuated Aβ-induced neurotoxicity (Costa et al., 2012). Moreover, exposure to Aβ caused a decrease in total and polymerized levels of beta-III tubulin and polymerized alpha-tubulin in mature hippocampal cells, which could be prevented by MK-801, memantine, and ifenprodil, suggesting a role for GluN2B-containing NMDARs in Aβ toxicity (Mota et al., 2012).

GluN2A and GluN2B are the major subunits of functional NMDARs. Some studies have suggested that GluN2A and GluN2B may differentially mediate NMDAR function at synaptic and extrasynaptic locations and play opposing roles in excitotoxicity (Thomas et al., 2006; Harris and Pettit, 2007). In the postsynaptic membrane of excitatory neurons, the density of NMDARs is higher in dendritic spines, within the postsynaptic density (PSD), considered synaptic NMDARs, than in the dendritic shaft and somatic membrane (Köhr, 2006). At the PSD, the receptors form a large macromolecular NMDAR complex (NRC), containing a vast collection of scaffolding, adaptor and effector proteins that are involved in activation of downstream signaling cascades and regulation of NMDAR function, membrane stability and trafficking (Sheng and Lee, 2000). Extrasynaptic NMDARs are localized at sites further from the PSD, on the spine neck, the dendritic shaft or soma (Newpher and Ehlers, 2008). Perisynaptic NMDARs are located on the plasma membrane (PM) within 200–300 nm of the PSD. The perisynaptic region may contain mobile receptors that are in transit to and from the PSD (Zhang and Diamond, 2006; Petralia et al., 2009). Synaptic and extrasynaptic NMDARs are gated by different endogenous co-agonists: D-serine for synaptic NMDARs and glycine for extrasynaptic NMDARs. Both co-agonists are of glial origin (Papouin et al., 2012). It was proposed that GluN2A- and GluN2B-containing receptors are predominantly found synaptically and extrasynaptically, respectively. For NMDAR redistribution plays a larger role during development, when there is a synaptic switch from GluN2B to GluN2A-containing receptors (Barria and Malinow, 2002). In addition, it has been found that synaptic and extrasynaptic NMDA receptors have opposing effects in determining the fate of neurons (Vizi et al., 2013). Calcium entry through synaptic GluN2A-containing NMDARs induces activity of cAMP response element binding protein (CREB) and gene expression of CREB-evoked brain-derived neurotrophic factor (BDNF), which is involved in the neuroprotective action of synaptic glutamatergic transmission. In contrast, Ca^2+^ entry through GluN2B-containing NMDARs, which are expressed extrasynaptically, triggers the CREB shut-off pathway (Leveille et al., 2008). There is some evidence that synaptic NMDARs support LTP, while extrasynaptic NMDARs mediate LTD in the mature brain (Massey, 2004). Specific NMDAR subunits are not confined to particular subcellular locations on PM. Some evidences shown that GluN2A- and GluN2B-subtypes are present at both synaptic and extrasynaptic sites (Thomas et al., 2006; Harris and Pettit, 2007; Martel et al., 2009; Zhou et al., 2013a). These suggest that any difference in signaling by synaptic and extrasynaptic NMDARs must be due to intracellular signaling pathways rather than subunit identity or mobility (Harris and Pettit, 2007). And some researchers thought that these results were partially refuted on the basis that these experiments were carried out on developing hippocampal cultured neurons; the same results may not be found under *in vivo* conditions (Newpher and Ehlers, 2008).

Certain interactions of NMDAR subunits with distinct signaling molecules may occur at synaptic but not at extrasynaptic sites (Köhr, 2006). Extrasynaptic NMDARs are exposed to ambient glutamate, whether this glutamate concentration is high enough to tonically activate extrasynaptic NMDARs remains controversial. Although microdialysis studies report that ambient glutamate concentrations *in vivo* are high enough to activate extrasynaptic NMDARs (Nyitrai et al., 2006), a study suggests that glutamate transporters regulate ambient glutamate concentrations at a level that is too low to cause significant receptor activation (Herman and Jahr, 2007). While, some reports that glutamate that is released into the extracellular space mainly from glial processes (Fellin et al., 2004) may result in the persistent activation of extrasynaptic GluN2B receptors, which are of high affinity and are sensitive to low concentrations of glutamate (Vizi, 2000).

Activation of synaptic NMDARs and large increases in [Ca^2+^]_i_ are required for LTP, whereas internalization of synaptic NMDARs, activation of extrasynaptic NMDARs and lower increases in [Ca^2+^]_i_ are necessary for LTD. LTP induction promotes recruitment of AMPARs and growth of dendritic spines, whereas LTD induces spine shrinkage and synaptic loss (Kullmann and Lamsa, 2007). Importantly, glutamate spillover from synapses or glutamate released from astrocytes activates extrasynaptic NMDARs (Fellin et al., 2004). Extrasynaptic NMDARs are activated not only at pathological situations (Hardingham et al., 2002), but also by bursts of activity that can occur under physiological situations (Harris and Pettit, 2008). Retinal ganglion cells express only extrasynaptic NMDARs and are invulnerable to NMDA neurotoxicity (Ullian et al., 2004). Synaptic NMDARs can also cause neurotoxicity (Sattler et al., 2000; Sinor et al., 2000) and can induce LTD (Malenka and Bear, 2004). Furthermore, Zhou et al. (2013b) demonstrate that activation of synaptic or extrasynaptic NMDAR alone stimulated pro-survival but not pro-death signaling, for they had overlapping but not opposing effects on genomic responses. Low-dose NMDA preferentially activated synaptic NMDAR and stimulated the extracellular signal-regulated kinase 1/2 (ERK1/2)-CREB-BDNF pro-survival signaling, while higher doses progressively activated increasing amount of extrasynaptic NMDAR along with synaptic NMDAR and triggered cell death program. While, Liu et al. (2007) suggested that the subunit composition of NMDARs rather than their cellular location determines the final effect of the activation of the NMDARs by glutamate.

*N*-methyl-D-aspartate receptors are highly mobile at both synaptic and extrasynaptic membranes. Importantly, the surface mobility of NMDARs appears to change with development (Harris and Pettit, 2007; Köhr, 2007) in a subunit composition-specific manner (Groc et al., 2006). GluN2A-containing NMDARs are less mobile than GluN2B-containing NMDARs, and the synaptic residency time of GluN2B-containing NMDARs decreases over development. This decrease in synaptic dwell time of GluN2B containing NMDARs is mediated by Reelin, an extracellular matrix protein (Groc et al., 2007). Regulatory mechanisms underlying synaptic and extrasynaptic NMDAR distribution include: protein–protein interactions, phosphorylation, palmitoylation, ubiquitination, role of proteases, such as calpain (Gladding and Raymond, 2011).

Aβ-induced synaptic depression may result from an initial increase in synaptic activation of NMDARs by glutamate, followed by synaptic NMDAR desensitization, NMDAR internalization, and activation of extrasynaptic or perisynaptic GluN2B-enriched NMDARs, which have a key role in LTD induction (Palop and Mucke, 2010). On one hand, Aβ downregulates the synaptic NMDAR response by promoting NMDAR endocytosis, leading to either neurotoxicity or neuroprotection. On the other hand, Aβ enhances the activation of extrasynaptic NMDARs by decreasing neuronal glutamate uptake and inducing glutamate spillover, subsequently causing neurotoxicity (Wang et al., 2013). Aβ oligomers bind with high affinity to cell surface cellular prion protein (PrP^C^). PrP^C^ and Fyn are enriched in PSD. Metabotropic glutamate receptor, mGluR5, has ability to couple Aβ-PrP^C^ with Fyn, controls the transmission of neurotoxic signals to intracellular substrates (Um et al., 2013; Haas et al., 2014). Aβ engagement of PrP^C^ activates Fyn to phosphorylate the GluN2B subunit of NMDARs, which was coupled to an initial increase and then a loss of surface NMDARs (Um et al., 2012). Pretreatment with the 6D11 antibody to PrP^C^ could prevented the Aβ-induced synaptic dysfunction (Um et al., 2012). Aβ also binds to the α7nAChR, resulting in increased Ca^2+^ influx, calcineurin activation and dephosphorylation and activation of STEP61. Increased STEP61 activity leads to Fyn inactivation and reduced NMDAR exocytosis, as well as enhanced GluN2B Y1472 dephosphorylation and increased NMDAR internalization (Kurup et al., 2010).

### Tau and NMDA Receptors

Tau is a major component of the NFT that represent a pathological hallmark of AD. In the healthy brain, tau is an exclusively axonal protein, engaged in the assembly and stability of microtubules. In contrast, in the AD brain, tau is hyperphosphorylated and forms fibrils that appear as neuropil threads in dendrites and as NFTs in the somatodendritic compartment and axons. It had provided strong evidence cerebral amyloid deposition precedes cerebral tau pathology in familial autosomal dominant AD, While, the appearance of NFTs precedes Aβ pathology in the vast majority of affected regions in sporadic AD (Morris et al., 2018). Roberson et al. (2007) first ruled out the possibility that tau reduction altered Aβ levels or aggregation, and uncoupled Aβ from downstream pathogenic mechanisms (Ittner et al., 2010).

Tau not only contributes to axonal structure by maintaining microtubule stability, but also is important in the regulation of synaptic function. Tau is required for Fyn-mediated NMDAR activation in the PSD (Ittner et al., 2010), and tau has been shown to be essential for the induction of LTD (Kimura et al., 2014) as well as BDNF-dependent morphological plasticity (Chen et al., 2012). Fyn, a member of the Src family of tyrosine kinases (Thomas and Brugge, 1997), can phosphorylate tau at its tyrosine 18 residue to generate pY18-tau (Lee, 2004) and can bind to tau through one or more proline-rich (PxxP) motifs in tau (Lee et al., 1998; Reynolds et al., 2008; Usardi et al., 2011). Fyn phosphorylates the NMDAR subunit GluN2B at Y1472 (Tezuka et al., 1999), which strengthens the interaction between NMDARs and PSD-95 in the PSD (Rong et al., 2001) and enhances the activity of GluN2B-containing NMDARs (Groveman et al., 2012). Some experiments indicated that tau is normally highly enriched in axons relative to dendrites (Binder et al., 1985), but in response to Aβ is extensively redistributed into the somato-dendritic compartment (Delacourte et al., 1990; Zempel et al., 2010). Excess Fyn accompanies the excess tau in AD dendrites and upregulates NMDA receptor activity there, flooding the dendrites with harmful levels of calcium. This calcium-driven excitotoxicity can damage postsynaptic sites and cause neuron death. Some results confirmed that glutamate-induced excitotoxicity is inhibited by reduction of tau (Roberson et al., 2007; Ittner et al., 2010) and exacerbated by overexpression of tau (Decker et al., 2016; Maeda et al., 2016). In turn, glutamate-induced excitotoxicity increased tau expression (Sindou et al., 1992; Esclaire et al., 1997) and phosphorylation (Sindou et al., 1994). Recently, it has been reported that activation of extrasynaptic NMDA receptors induces tau overexpression, with a simultaneous neuronal degeneration and decreased neuronal survival (Sun et al., 2016).

### Some Unconventional NMDAR Signaling in AD

#### Presynaptic NMDAR

Traditionally, NMDARs are thought to be located at the postsynaptic membrane, while recent anatomical and physiological evidence demonstrates that they may also exist at presynaptic terminals. Presynaptic NMDARs (preNMDARs) can regulate presynaptic glutamate release, and reshape synaptic transmission and plasticity (Corlew et al., 2007; Paoletti et al., 2013; Banerjee et al., 2016; Dore et al., 2017). Therefore, preNMDAR subunit composition is critical for modulating where and how preNMDARs influence glutamate release. The subunit composition of preNMDARs shows strong variability, depending on the brain region, all four GluN2 subunits (Glu2A-D) as well as GluN3A can be incorporated (Bouvier et al., 2015). The presynaptic GluN2B subunit has been found in many brain regions, such as the hippocampus (Jourdain et al., 2007; McGuinness et al., 2010; Berg et al., 2013), the cerebellum (Rossi et al., 2012), the entorhinal cortex (Yang et al., 2006, 2008), somatosensory (Brasier and Feldman, 2008) and visual cortex (Banerjee et al., 2016). Although appears and peaks later during development (Monyer et al., 1994; Henson et al., 2010), GluN2A subunit can also be incorporated into preNMDAR sites (Bouvier et al., 2015). At cerebellar parallel fiber-Purkinje cell synapses, preNMDARs are predominately GluN1/GluN2A diheteromeric (Bidoret et al., 2009). Furthermore, GluN2B and GluN3A subunits, likely combining into triheteromeric GluN1/GluN2B/GluN3A, are essential preNMDARs in the developing L2/3 visual cortex (Li et al., 2009; Larsen et al., 2014).

It has been reported that preNMDARs may regulate both spontaneous and evoked release. Although the two forms of release were initially thought to employ the same machinery, the emerging evidence indicates that preNMDARs control evoked and spontaneous release by distinct mechanisms. PreNMDARs may control spontaneous release independently of Mg^2+^ and Ca^2+^ while regulating evoked release in a frequency-dependent manner by relying on the more conventional Mg^2+^-dependent pathway (Kunz et al., 2013; Paoletti et al., 2013; Abrahamsson et al., 2017; Bouvier et al., 2018). Abrahamsson et al. (2017) showed that preNMDARs in L5 pyramidal cells regulate evoked and spontaneous release via RIM1αβ and JNK2-dependent pathway, respectively.

In addition, the activation of preNMDARs is necessary for the induction of LTD (Duguid, 2013; Paoletti et al., 2013), but it is noteworthy that preNMDARs roles shift over development. Induction of LTD in visual cortex pyramidal cells of young mice (before postnatal 20 days) requires activation of presynaptic, whereas in older mice, the induction of LTD requires postsynaptic NMDARs activation (Corlew et al., 2007). Contrast to this results, Carter and Jahr (2016) show that in the somatosensory cortex of 2- to 3-week-old rats and mice, it is postsynaptic, not preNMDARs that required for LTD induction. This contradictory result may be due to different brain regions observed. Not only being required for the induction of LTD, preNMDARs also involve in LTP induction (McGuinness et al., 2010). Park et al. (2014) reported that preNMDARs play a pivotal role in the induction of LTP at mouse corticostriatal synapses. Activation of preNMDARs induces BDNF secretion via enhancing Ca^2+^ signals in the axonal terminals, indicating that preNMDARs are equally essential as postsynaptic NMDARs in LTP induction (Park et al., 2014).

Bell et al. (2007) found that subjects with mild cognitive impairment (MCI) displayed a paradoxical elevation in glutamatergic presynaptic bouton density, which then depletes and drops with disease progression. These results pointed out that dystrophic neurite generation and reduced presynaptic bouton densities detrimentally influence neurotransmission and cognitive function in later stages of AD. Although some advances have been made, much work still need to clarify the precise functions and molecular mechanisms on preNMDAR.

#### Glial NMDAR

While neuronal NMDARs have been widely studied, NMDARs are also expressed in many non-neuronal cells, including astrocytes. Compared to neuronal receptors, astrocytic NMDA receptors are poorly understood. The emerging evidence suggests that astrocytic NMDARs have distinct structural and functional properties, including weak susceptibility to Mg^2+^ blockade and less Ca^2+^ permeability (Hogan-Cann and Anderson, 2016). NMDAR expression and function in astrocytes has been demonstrated in cultured astrocytes and mouse neocortex (Lalo, 2006; Palygin et al., 2011; Montes and Aguilera, 2015; Li et al., 2016). In human primary astrocytes, all seven identified NMDAR subunits (GluN1, GluN2A-D, GluN3A-B) were found. Increasing data demonstrate that astrocytes express NMDARs with a triheteromeric configuration combining GluN1, GluN2C or D, and a GluN3 subunit (Burzomato et al., 2010; Henson et al., 2012). Glutamate and QUIN both could activate astrocytic NMDARs, which in turn enhances Ca^2+^ influx and induces signaling cascade (Lee et al., 2010). It is established that astrocytic functional NMDARs are able to respond to neuronal glutamatergic input, which accompany with dynamic intracellular Ca^2+^ rise triggering gliotransmitter-mediated regulation of synapses function (Kato et al., 2006; Lalo, 2006; Hogan-Cann and Anderson, 2016). Astrocytic NMDARs may also be involved in neuroinflammatory processes, and contribute to morphological transformations characteristic of reactive astrogliosis and mediate the release of proinflammatory cytokines (Sofroniew, 2009; Ting et al., 2009; Gerard and Hansson, 2012). Notably, astrocytic NMDARs maybe contribute to AD due to their roles in facilitating glutamate excitotoxicity (Lee et al., 2010; Mota et al., 2014). Our research found that Aβ-induced early synaptotoxicity can be exacerbated after treatment with antagonism of astrocytic GluN2A and GluN2B, and nerve growth factor (β-NGF) is may be as a mediator in the synaptoprotection of astrocytic GluN2 activation (Li et al., 2016). In co-cultures system, pre-treatment of astrocytes with 1 μM or 10 μM NMDA to activate GluN2A or GluN2B, before exposure to Aβ_1-40_, was found to prevent the Aβ introduced PSD-95 and synaptophysin decreased. While blockade of astrocytic GluN2A with TCN-201 or GluN2B with ifenprodil respectively both aggravated the synaptotoxic effects of Aβ.

Additionally, NMDARs are also expressed by oligodendrocyte lineage cells, as mediators of intracellular Ca^2+^ accumulation, leading to reduced oligodendrocyte survival and white matter damage (Káradóttir et al., 2005; Salter and Fern, 2005; Micu et al., 2006). The dominant force behind NMDA-induced currents in mature oligodendrocytes is actually elevated extracellular K^+^ released upon activation of neuronal or astrocytic NMDARs. Increased in oligodendrocytic Ca^2+^ would be gated by transient receptor potential cation channel (TRP) A1 (Hamilton et al., 2016). Oligodendrocyte precursor cell (OPC) NMDARs may also contribute to myelination. Activation of NMDARs in OPC cultures increased migration (Wang et al., 1996), myelin basic protein expression (Wake et al., 2011) and differentiation (Li et al., 2013).

Rather surprisingly, a recent study observed that NMDARs were present in primary cultures of microglia from mice cortex and hippocampus (Franco et al., 2018), and exposure to NMDAR co-agonists resulted in evoked inward currents and intracellular Ca^2+^ increases sensitive to inhibition by the non-competitive NMDAR channel blocker, MK801 (Murugan et al., 2011; Kaindl et al., 2012). NMDAR activation in microglia leads to significant ERK1/2 phosphorylation. ERKs phosphorylation, namely, NMDAR interact with and signaling to mitogen-activated protein kinase (MAPK) depends on CaM and NCS1 in neurons, whereas the signaling via NMDAR in microglia only depends on CaM. NMDAR function was potentiated in microglia from the APP_Sw,Ind_ transgenic mice, indicating that the NCS1–NMDAR interaction is relevant for receptor function in the microglia of the AD mouse model (Franco et al., 2018).

#### Metabotropic NMDAR

It was thought that NMDAR-dependent synaptic plasticity was controlled entirely by Ca^2+^ influx, and the increased cytoplasmic Ca^2+^ acts as a second messenger in the postsynaptic neuron (Dore et al., 2017). More recently provocative emerging evidence assumes that, upon binding of glutamate, NMDARs can produce long-term changes in synaptic function in the absence of calcium conductance (Chung, 2013; Nabavi et al., 2013; Birnbaum et al., 2015; Stein et al., 2015). In other words, NMDAR can act as a metabotropic receptor and signal metabotropically, without the need for Ca^2+^ influx through the channel (Kessels et al., 2013; Nabavi et al., 2013; Stein et al., 2015; Hell et al., 2016; Sanders et al., 2018).

A conventional view proposes that NMDARs triggers LTP via a high level of Ca^2+^ influx, while metabotropic receptor arises from synaptic depression induced by low frequency stimulation (LFS), which is called as NMDAR-dependent LTD (Malenka, 1994; Thiels et al., 1996). Recent results demonstrated that glutamate binding alone is sufficient to induce conformational changes of NMDARs, triggering p38MAPK signaling cascades, and in turn to induce LTD (Nabavi et al., 2013; Carter and Jahr, 2016; Hell et al., 2016). It is noteworthy that Ca^2+^ rise could induce LTP; while keeping at the baseline level of intracellular Ca^2+^ is necessary for metabotropic NMDAR activation, which leads to synaptic depression (Chung, 2013).

Consistent with this metabotropic signaling, some researchers found preNMDARs may control spontaneous release without need of Mg^2+^ and Ca^2+^, while evoked release was sensitive to Mg^2+^ (Kunz et al., 2013; Abrahamsson et al., 2017). PreNMDARs promote transmitter release partly via protein kinase C signaling (Kunz et al., 2013). These data suggest that preNMDARs may signal metabotropically, and further support the emerging propose that evoked and spontaneous release are through distinct machinery (Kavalali, 2015; Dore et al., 2017). Although mounting findings support an ion flux-independent mechanism for NMDAR-dependent LTD (Stein et al., 2015; Carter and Jahr, 2016; Wong and Gray, 2018), it has been challenged by some recent discoveries (Babiec et al., 2014; Volianskis et al., 2015; Sanderson et al., 2016). It is more possible that two distinct forms of NMDAR-dependent LTD co-exist indeed: one requires ion-flux and the other does not (Dore et al., 2017).

In addition, some studies demonstrated that astrocytic NMDARs may also act through metabotropic signaling pathways (Jimenez-Blasco et al., 2015; Montes and Aguilera, 2015), in which a phospholipase C-mediated endoplasmic reticulum Ca^2+^ rise and activation of protein kinase Cδ may be involved (Jimenez-Blasco et al., 2015), but much work remains to clarify the complex mechanism (Hogan-Cann and Anderson, 2016).

Early synaptic dysfunction in AD is associated with increased levels of Aβ oligomers, which causes a rapid NMDAR-dependent synaptic depression and spine loss (Shankar et al., 2007; Dore et al., 2017). While, some studies showed that Aβ-induced NMDAR-dependent synaptic depression did not require ion flux through the receptor (Kessels et al., 2013; Tamburri et al., 2013; Birnbaum et al., 2015), and was blocked by AP-5, but not MK-801, suggesting a metabotropic effect of NMDARs contributes to Aβ-induced synaptic dysfunction. It may be a common mechanism between metabotropic NMDAR-dependent LTD and Aβ-induced synaptic depression (Hell et al., 2016; Foster et al., 2017).

## Treatment

The current therapeutic arsenal of AD comprise of two classes of medications: the cholinesterase inhibitors (ChEIs), which include galantamine and rivastigmine (both approved for use in mild to moderate AD) and donepezil (approved for use in mild to severe AD); and the non-competitive NMDA receptor antagonist memantine (approved for use in moderate to severe AD). Combining ChEIs and memantine could offer greater benefits on behavior, cognition, and global outcomes (Patel and Grossberg, 2011; Deardorff and Grossberg, 2016). However, these drugs provide only symptomatic benefits in AD (Salomone et al., 2012). Any CNS disorder in which neuronal loss is related to glutamate-induced excitotoxicity has the potential to be treated by the inhibition of NMDARs. Phencyclidine, ketamine, MK-801 (dizocilpine) and memantine all target NMDARs. But due to severe side effects of phencyclidine, ketamine, and MK801, such as high affinity for and long dwell time on the receptor, their clinical application is hindered (Ellison, 1995). By contrast, memantine has lower affinity and is well tolerated, thus is used to treat moderate to severe AD (Reisberg et al., 2003; Pierson et al., 2014; Song et al., 2018). Inspiringly, riluzole, a new inhibitor of glutamate release and postsynaptic glutamate receptor signaling, is now in a phase II trial in mild AD patients (Graham et al., 2017).

Memantine has an inhibitory effect on NMDAR-mediated excitotoxicity (Parsons et al., 2007), strong voltage dependency, and rapid unblocking kinetic properties. Similar to Mg^2+^, it blocks the NMDAR and does not leave it in the presence of tonic pathological over-activation of the receptor, while allowing the transmission of transient physiological signals crucial for memory and learning processes (Danysz and Parsons, 2012). It has been shown that memantine increases the release of neurotrophic factors from astroglia (Wu et al., 2009), which may account for its survival effect, and exerts a neuroprotective effect. To date, memantine is the only NMDAR antagonist that is clinically approved and preferentially acts as an antagonist of non-synaptic NMDARs (Lipton, 2006). Extrasynaptically located NMDARs have a relevant role in neurodegeneration. Activation of extrasynaptic receptors may not only regulate expression of tau (Paterlini et al., 1998), but also induce transcriptional inactivation of CREB (Rönicke et al., 2011; Grochowska et al., 2017) and also contribute to early synaptic dysfunction (Franco et al., 2018). The low-affinity antagonist memantine will be the focus of growing interest in targeting extrasynaptic GluN2B-containing NMDARs. In addition, selective enhancement of synaptic activity by low doses of NMDA, or reduction of extrasynaptic activity by memantine, halts Aβ-induced neurotoxicity. The concomitant blockade of GluN2A-containing receptors impaired the activation of protective pathways. In contrast, the selective inhibition of GluN2B-containing NMDARs may be beneficial because it prevents neuronal cell death but leaves the protective pathways intact. Therefore, it is almost certain that in the future, neuroprotective drugs for AD should aim at both the enhancement of synaptic activity and the disruption of extrasynaptic GluN2B-containing NMDAR-dependent death signaling (Wang et al., 2013), the non-selective NMDA antagonists cannot be effective as neuroprotective agents.

The first GluN2B-selective drug is ifenprodil, which inhibits GluN2B receptor-mediated currents with an IC50 of 0.34 μM, whereas its affinity for GluN2A receptors is 400-fold lower, with an IC50 of 146 μM (Williams, 1993). Ifenprodil binds to the N-terminal lysine/isoleucine/valine-binding protein (LIVBP)-like domain of GluN2B and acts as a non-competitive antagonist in a use-dependent and voltage-independent manner. There have been several second generation ifenprodil analogs, including CP-101,606 (traxoprodil) and Ro 25-6981 (Table 1), that exhibit a much greater selectivity for GluN2B subunits. Indole-2-carboxamide derivatives, such as RG-13579 and RG-1103, are a new group of GluN2B-selective antagonists. RG-13579 and RG-1103 bind GluN2B-containing receptors 1,600- and 13,600-fold more selectively, respectively, than they bind GluN2A-containing receptors (Nagy, 2004). Inhibition of the interactions of GluN2B with Fyn and GluN2B tyrosine phosphorylation may contribute to the CP-101,606-induced downregulation of NMDAR function (Kong et al., 2015). *In vivo* [^3^H]MK-801 binding study shows that NMDAR activity in the rodent forebrain can be inhibited completely by channel blockers, AZD6765 (lanicemine) and MK-801, but only partially (∼60%) by GluN2B receptor antagonists, CP-101,606, MK-0657 (CERC-301), EVT-101, Ro 25-6981 and radiprodil, at doses that completely occupied GluN2B receptors (Fernandes et al., 2015). Graef et al. (2015) demonstrated that a single dose of either the non-selective NMDA receptor blocker ketamine or the selective GluN2B antagonist CP-101,606 can enhance *ex vivo* hippocampal LTP in rats 24 h after treatment.

**Table 1:** Various classes of NMDAR antagonists.

Mechanism of NMDAR antagonists	Examples	IC_50_	Key reference
**GluN2B**			
Non-competitive	Ifenprodil	0.34 μM	Williams, 1993
	CP-101,606	10 nM	Chenard et al., 1995
	Ro 25-6981	0.003 μM	Fischer et al., 1997
**GluN2A**			
Non-competitive	Zinc	5.0 ± 1.6 nM	Chen et al., 1997
**GluN2D**			
	NAB-14	580 nM	Swanger et al., 2017
**GluN3**			
Non-competitive	TK13	67 μM (GluN3A) 49 μM (GluN3B)	Kvist et al., 2013
	TK30	14 μM (GluN3A) 7.4 μM (GluN3B)	Kvist et al., 2013
**GluN3B**			
Competitive	TK80	79 μM	Kvist et al., 2013


Zinc binds to the leucine/isoleucine/valine binding protein (LIVBP)-like domain of GluN2A, displays a greater than 50-fold selectivity for GluN1/GluN2A over GluN1/GluN2B receptors (Paoletti et al., 1997). GluN2A-selective negative allosteric modulator (NAM) bound LBD heterodimer, corresponding to active and inhibited receptor states reveal a molecular switch in the modulatory binding site that mediate the allosteric inhibition (Yi et al., 2016). Zinc and ifenprodil bind with high affinity to the ATDs of GluN2A and GluN2B, respectively (Zhu and Paoletti, 2015). In hippocampal synapses, zinc decreased the EPSC peak and prolonged the deactivation. Ifenprodil, in contrast, decreased the peak but did not prolong the deactivation (Tovar and Westbrook, 2017).

Binding sites of GluN2A-NTD (N-terminal domain) and GluN2B-NTD retain selective high affinity for their ligand, zinc and ifenprodil. However, each ligand produces only partial inhibition, and maximal inhibition requires occupancy of both GluN2-NTDs by their respective ligands (Hatton and Paoletti, 2005). With regard to the triheteromeric NMDARs, such as GluN1/GluN2A/GluN2B, a single GluN2A or GluN2B subunit is sufficient to confer high affinity to zinc or ifenprodil, but the maximal level of inhibition is greatly reduced compared with diheteromeric NMDARs (Paoletti, 2011). Similarly, GluN1/Glu2A/GluN2C receptors are inhibited by zinc with high potency but low efficacy. Therefore, interactions between homologous NTDs determine the unique pharmacological properties of triheteromeric NMDARs (Paoletti, 2011). The sensitivity of triheteromeric NMDARs to zinc or ifenprodil provides an example of intermediate pharmacology, with reduced potency and efficacy (Hansen et al., 2014; Stroebel et al., 2014).

A recent work identifies a new series of NAMs to study GluN2C and GluN2D function in brain circuits. The prototypical compound, NAB-14, is >800-fold selective for recombinant GluN2C/GluN2D over GluN2A/GluN2B in Xenopus oocytes and has an IC_50_ value of 580 nM at recombinant GluN2D-containing receptors expressed in mammalian cells, which inhibition reside in the GluN2D M1 transmembrane helix. NAB-14 inhibits GluN2D-mediated synaptic currents in rat subthalamic neurons and mouse hippocampal interneurons, but has no effect on synaptic transmission in hippocampal pyramidal neurons, which do not express GluN2C or GluN2D (Swanger et al., 2017). Perszyk et al. (2016) data provide evidence that hippocampal interneurons contain synaptic NMDARs possessing a GluN2D subunit, which can influence interneuron function and signal processing. These works suggest that selectively modulating brain circuits involving the GluN2C and GluN2D subunits is likely to be developed into clinical therapies (Swanger et al., 2017).

GluN3A subunit was cloned almost two decades ago (Ciabarra et al., 1995; Sucher et al., 1995), followed by cloning of the GluN3B subunit in the beginning of this century (Andersson et al., 2001; Nishi et al., 2001). The GluN3 subunits appears to function as modulatory subunits that reduce the susceptibility of NMDA receptors to Mg^2+^-blockage and reduce Ca^2+^-permeability (Perez-Otano et al., 2001; Cavara et al., 2010). Furthermore, GluN3 subunits are involved in synapse maturation (Henson et al., 2012), synaptic plasticity (Larsen et al., 2011), and neuroprotective (Martínez-Turrillas et al., 2012). Overexpression of GluN3A might attenuate NMDAR mediated cell death by reducing Ca^2+^ permeability of existing NMDARs (Nakanishi et al., 2009). Consistent with the idea that GluN3A might offer neuroprotective benefits, high levels of GluN3A expression during early brain development might explain why excitotoxicity is not more prevalent at ages before the maturation of inhibitory circuitry. GluN3A dysfunction may contribute to neurological disorders involving NMDARs, and the subunit offers an attractive therapeutic target given its distinct pharmacological and structural properties. The study of GluN3A pharmacology is still in its infancy. However, it is clear that GluN3A shares more commonalities with GluN1 than it does with GluN2 subunits. The GluN3A NTD constitutes a crucial regulatory determinant of GluN1/GluN3A receptor function. A hemophilic intersubunit interaction amongst the GluN3A NTDs and the transition region between GluN3A NTD and LBD constitute major structural determinants underlying the low efficacy of glycine-gated GluN1/GluN3A receptors (Mesic et al., 2016). Glycine binds GluN3A with much higher affinity than it binds GluN1, rodent GluN3A, *K*_d_ = 40 nM, 650 times less than that for GluN1 (Yao, 2006), and human GluN3A, *K*_d_ = 535 nM (Nilsson et al., 2007). D-serine also binds human and rat GluN3A with high affinity (Yao, 2006; Nilsson et al., 2007). Very little is known about how GluN3A-containing receptors can be pharmacologically blocked. Kvist et al. (2013) have succeeded in identifying one novel competitive antagonist, identified 6-hydroxy-[1,2,5] oxadiazolo [3,4-b] pyrazin-5(4H)-one (TK80), with preference for the GluN3B subunit. In addition, they serendipitously identified two novel antagonists, [2-hydroxy-5-((4-(pyridin-3-yl) thiazol-2-yl) amino] benzoic acid (TK13) and 4-(2,4-dichlorobenzoyl)-1H-pyrrole-2-carboxylic acid (TK30), that appear to be non-competitive GluN3 antagonists (Kvist et al., 2013). When GluN1/GluN3A diheteromers are expressed in heterologous systems, they exhibit little electrophysiological block by the classic NMDAR antagonists APV, MK-801, or memantine (Chatterton et al., 2002). Interestingly, GluN1/GluN2/GluN3A triheteromeric receptors may also be blocked by antagonists targeting GluN2 subunits, as receptors composed of GluN1/GluN2B/GluN3A triheteromers and GluN1/GluN2B diheteromers are similarly blocked by high concentrations of the GluN2B antagonist ifenprodil (Smothers and Woodward, 2003). One might predict that, compared to GluN1/GluN2B diheteromers, GluN1/GluN2B/GluN3A triheteromers might be less sensitive to lower concentrations of ifenprodil where GluN2B specificity is greater, as previous studies have shown that the magnitude of ifenprodil block depends on the number of GluN2B subunits contained within the NMDAR complex (Hatton and Paoletti, 2005).

Death-associated protein kinase 1 (DAPK1), Ca/calmodulin (CaM)-dependent protein kinase II α (CaMKIIα), and protein kinase A (PKA) are able to phosphorylate NMDARs. A novel approach for preventing neurotoxicity is to inhibit the phosphorylation of GluN2B-containing NMDARs by CaMKIIα. The CaMKIIα antagonist KN-93, a methoxybenzene sulfonyl derivative that competitively inhibits calmodulin binding to CaM kinase with a K_i_ of 0.37 μM (Sumi et al., 1991), reduces the effect of CaMKIIα-mediated GluN2B phosphorylation (Farinelli et al., 2012). Protein phosphatase 1 (PP1), highly abundant protein in nerve cells, which co-localizes with the GluN2B subunits of NMDARs, is able to dephosphorylate the Ser1303 residue of GluN2B-containing NMDARs and prevent CaMKII from phosphorylating the receptors, thereby preventing Ca^2+^ overload inhibiting neurodegeneration (Raveendran et al., 2009; Farinelli et al., 2012; Vizi et al., 2013). Additionally, in the culture cortical neurons, treatment with 17-β-estradiol (E2) and estradiol receptor, the G-protein-coupled receptor 30 (GPR30) agonist G1 (K_i_ of 11 nM) attenuated the excitotoxicity induced by NMDA exposure. The short-term activation of GPR30 does not affect the expression of either GluN2A- or GluN2B-containing NMDARs; however, it depresses GluN2B subunit phosphorylation at Ser-1303 by inhibiting the dephosphorylation of DAPK1. The neuroprotection mediated by GPR30 is dependent on G-protein-coupled signals and ERK1/2 activation (Liu et al., 2012). Studies have shown that tricyclic desipramine and the serotonin-selective reuptake inhibitor (SSRI) fluoxetine also inhibit NMDA-induced currents in cortical cell cultures. Fluoxetine is a selective inhibitor of GluN2B-containing NMDARs (concentration that causes 50% inhibition, IC_50_ = 9.74 μM), whereas desipramine inhibits both GluN1/GluN2A (IC_50_ = 5.10 μM) and GluN1/GluN2B (IC_50_ = 3.61 μM) subtypes (Kiss et al., 2012). Szasz et al. (2007) observed that inhibition by desipramine (IC_50_ = 3.13 μM) was voltage-dependent, and the drug was unable to bind to NMDARs that were blocked by Mg^2+^, whereas the inhibition by fluoxetine (IC50 = 10.51 μM) was voltage-independent, and an association with the NMDAR was still possible in the presence of a Mg^2+^ block (Yux et al., 1996; Bolo, 2000).

A recent work suggest that the drugs targeting NMDA receptor function or downstream signaling cascades, can restore network and may be effective in treating AD (Zong et al., 2016). At the synapse, the NMDA receptor subunits combined with the post-synaptic related proteins (such as PSD-95) to form a macromolecular complex, which is central for mediating receptor-activated signaling cascades; thus a strategy uncoupling the NMDA receptor from PSD-95 would eliminate excitotoxicity (Morimoto, 2018). It is known that Fyn phosphorylating the GluN2 facilitate interaction of the NMDAR complex with PSD-95 (Nakazawa et al., 2001), linking NMDARs to excitotoxic downstream pathway (Salter and Kalia, 2004). It has been demonstrated that disruption of this interaction prevents excitotoxicity without affecting synaptic NMDA currents (Aarts et al., 2002). Some studies have suggested that agonist binding to NMDARs can signal cascade independently of Ca^2+^ influx through the receptor. These findings may lead to new pharmacological approaches to target specific synaptic signaling pathway in AD (Hell et al., 2016). Therefore, there is an urgent need to develop novel pharmacological compounds selectively targeting specific receptor subunits and the various signaling pathways (Paoletti et al., 2013).

## Conclusion and Perspectives

As Alzheimer’s progresses, brain cells die and synapses are lost, causing cognitive symptoms to worsen. All of the prescription medications currently approved to treat Alzheimer’s symptoms in early to moderate stages are from a class of drugs called ChEIs. The non-competitive NMDA receptor antagonist memantine and a combination of memantine and donepezil are approved by the United States Food and Drug Administration (FDA) for treatment of moderate to severe AD. While current medications cannot stop the damage Alzheimer’s causes to brain cells, they may help lessen or stabilize symptoms for a limited time. The researches focusing mainly on Aβ amyloid deposits and tau protein aggregates forming NFTs have yielded disappointing results. As such, there is a need to develop effective and safe disease modifying treatments that directly target AD pathology and alter the course of AD progression (Ruthirakuhan et al., 2016). It has been motivating researchers to study drugs in earlier stages of the disease, particularly in pre-clinical AD, even before MCI, instead of mild-to-moderate AD trials (Khoury et al., 2017).

AD, related to glutamate-induced excitotoxicity, has been treated by the inhibition of NMDARs. However, clinical trials that have applied high affinity NMDA antagonists have so far failed. This failure has been due to a lack of efficacy and unexpected paradoxical side effects (Ellison, 1995). These negative results can be explained by the opposing role of the different subtypes of NMDARs, and all subtypes are inhibited by the administered drugs. The heteromeric nature of NMDARs provides a wide variety of receptor subtypes, allowing for a rich diversity in receptor signaling properties. While synaptic GluN2A-containing NMDARs activation is involved in neuroprotection, the stimulation of extrasynaptic GluN2B-containing NMDARs triggers cell destruction pathways. The findings that GluN2A- and GluN2B-containing NMDARs can be selectively inhibited by antagonists may be useful in determining pharmacological targets for drug development. In addition, the sensitivity of triheteromeric NMDARs to zinc or ifenprodil with reduced potency and efficacy (Hansen et al., 2014; Stroebel et al., 2014). It will require the generation of NMDAR antagonists that are highly efficient and highly selective (Köhr, 2006). Modern research is trying to discover effective disease-modifying therapies, which specifically target the pathophysiologic signaling, to delay onset and progression of the disease (Tayeb et al., 2012). In future, continuous and sustained efforts are still needed before NMDAR-targeted interventions can transform into effective treatments (Paoletti et al., 2013).

## Author Contributions

JL and LC wrote most part of the review. YS and HL searched the references and participated in the writing. YW designed the review and revised it.

## Conflict of Interest Statement

The authors declare that the research was conducted in the absence of any commercial or financial relationships that could be construed as a potential conflict of interest.
